# Porcine circovirus type 2 upregulates endothelial-derived IL-8 production in porcine iliac artery endothelial cells via the RIG-I/MDA-5/MAVS/JNK signaling pathway

**DOI:** 10.1186/s12917-020-02486-1

**Published:** 2020-07-29

**Authors:** Fengyang Shi, Qiuming Li, Shiyu Liu, Fengying Liu, Jianfang Wang, Defeng Cui, Xiaolin Hou, Shuanghai Zhou, Yonghong Zhang, Huanrong Li

**Affiliations:** 1 College of Animal Science and Technology, Beijing University of Agriculture, No. 7 Beinong Road, Beijing, 102206 Changping District China; 2 Beijing Key Laboratory of TCVM, Beijing University of Agriculture, No. 7 Beinong Road, Beijing, 102206 Changping District China

**Keywords:** Porcine circovirus type 2, Endothelium-derived IL-8, Porcine iliac artery endothelial cells, RIG-I-like signaling pathway, JNK signaling pathway

## Abstract

**Background:**

Dysfunction of endothelial cells and vascular system is one of the most important pathological changes of porcine circovirus disease (PCVD) caused by porcine circovirus type 2 (PCV2). PCV2-infected endothelial cells can upregulate the production of endothelial-derived IL-8, which can inhibit the maturation of dendritic cells. Endothelial-derived IL-8 has different structural and biological characteristics compared with monocyte-derived IL-8. However, the mechanism of endothelial-derived IL-8 production is still unclear.

**Results:**

Key molecules of RIG-I-like signaling pathway RIG-I, MDA-5, MAVS and a key molecule of JNK signaling pathway c-Jun in PCV2-infected porcine iliac artery endothelial cells (PIECs) were upregulated significantly detected with quantitative PCR, Western blot and fluorescence confocal microscopy, while no significant changes were found in NF-κB signaling pathway. Meanwhile, the expression of endothelial-derived IL-8 was downregulated after RIG-I, MDA-5, or MAVS genes in PIECs were knocked down and PIECs were treated by JNK inhibitor.

**Conclusions:**

PCV2 can activate RIG-I/MDA-5/MAVS/JNK signaling pathway to induce the production of endothelial-derived IL-8 in PIECs, which provides an insight into the further study of endothelial dysfunction and vascular system disorder caused by PCV2.

## Background

Porcine circovirus type 2 (PCV2) is a major pathogen associated with postweaning multisystemic wasting syndrome (PMWS), which is characterized by lymphocyte apoptosis and inflammatory cell infiltration [1]. In addition, endothelial cell disfunction and vascular system disorders are also the main symptoms of porcine circovirus disease (PCVD) [2]. The PCV2-infected endothelial cells can regulate leukocyte migration, inflammatory responses [3] and induce the production of endothelial-derived IL-8 [4]. It is reported that endothelial-derived IL-8, with a pentapeptide AVLPR extension at the NH2-terminus [5], has different biological function compared with monocyte-derived IL-8 [6]. Our previous reports also showed that the endothelial IL-8 upregulated by PCV2 infection could inhibit the maturation and antigen presentation of dendritic cells [4, 7].

Epithelial IL-8 can be induced by parainfluenza virus type 1 via phosphorylation (p38) of the mitogen-activated protein kinase (p38-MAPKs) signaling pathway [8]. Monocyte-derived IL-8 can be induced by PCV2 via the TLR2/MyD88/NF-κB signaling pathway in porcine alveolar macrophages [9]. Some cytokines can be induced by pattern recognition receptors (PRRs), such as retinoic acid-inducible gene I (RIG-I) and melanoma differentiation-associated gene-5 (MDA-5) [10, 11], which can recruit downstream molecules (NF-κB, ERK, JNK, or PI3K-Akt) by binding to mitochondrial antiviral signaling protein (MAVS) [12–14]. It is reported that PCV2 can induce IFN-β production via the RIG-1/MDA-5/MAVS/IRF signaling pathway in PK-15 cell [15]. Classical swine fever virus can induce the production of IL-8 and other proinflammatory cytokines via the RIG-I/MDA-5 pathway [16, 17]. But whether the mechanism of endothelial-derived IL-8 production is consistent to that of other cell-derived IL-8 is still unknown.

Here, the study focused on the signal pathway of endothelial-derived IL-8 production in PIECs, providing a basis for the further study of endothelial dysfunction and vascular system disorder caused by PCV2.

## Results

### PCV2 induces RIG-I, MDA-5 and MAVS activation in PIECs

**Fig. 1:**
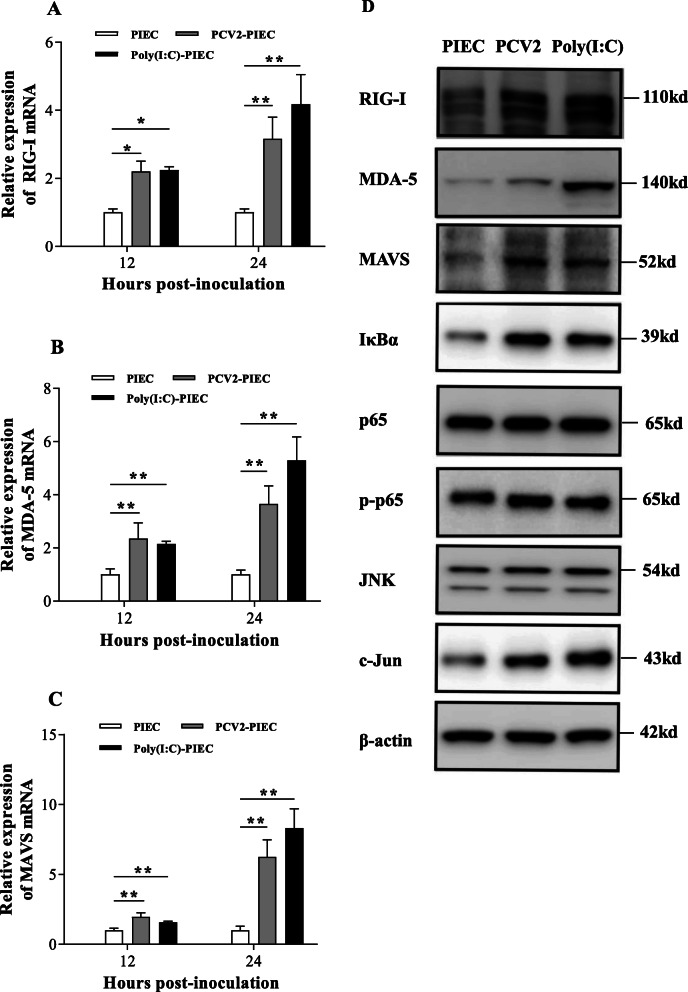
The RIG-I, MDA-5, and MAVS mRNA expression levels induced by PCV2 and the changes of all related protein expression after PCV2 infection in PIECs. PIECs were infected with PCV2 (MOI = 0.5) for 1 h and then cultured for 12 and 24 h. The levels of RIG-I (**a**), MDA-5 (**b**) and MAVS (**c**) mRNA expression levels were assessed by qPCR. The uninfected PIEC was used as a negative control and Poly(I:C) treatment was used as a positive control. The data are results of three independent experiments of relative expression values compared with β-actin mRNA, and represented as the mean and standard deviation (error bars) for each group. *, *P* < 0.05. **, *P* < 0.01. PIECs were collected after PCV2 (MOI = 0.5) infection for 24 h. Cell lysates were examined by Western blot with anti-RIG-I, anti-MDA-5, anti-MAVS, anti-IκBα, anti-NF-κB p65, anti-NF-κB p-p65, anti-JNK and anti-c-Jun antibodies (**d**). The uninfected PIEC was used as a negative control, Poly(I:C) treatment was used as a positive control and endogenous β-actin expression was used as an internal control

**Fig. 2:**
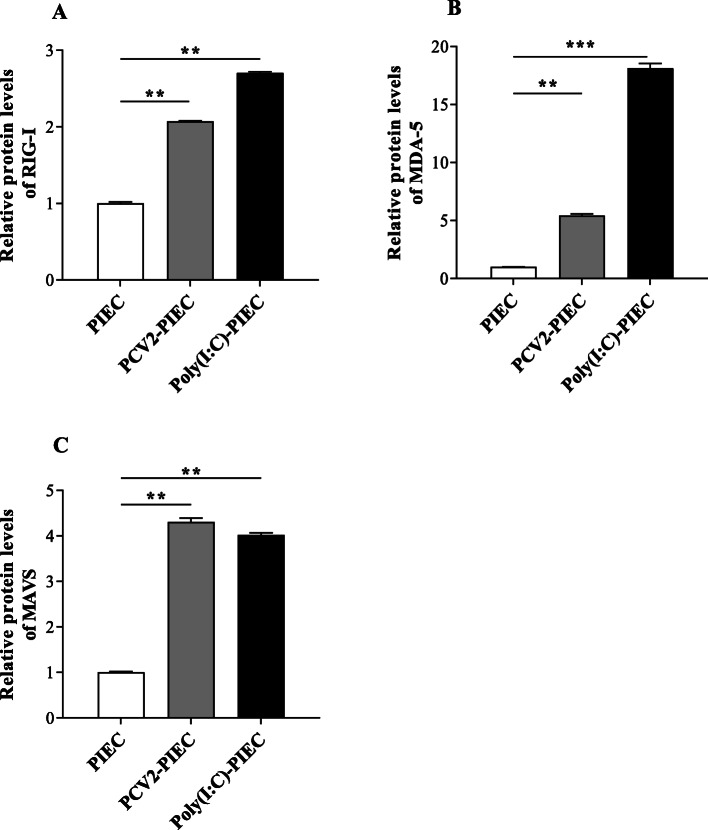
The RIG-I, MDA-5 and MAVS protein relative expression levels induced by PCV2 in PIECs. Quantitative analyses of the immunoblot data of RIG-I (**a**), MDA-5 (**b**) and MAVS (**c**) was performed. The uninfected PIEC was used as a negative control and Poly(I:C) treatment was used as a positive control. Data are results of three independent experiments and presented as the mean and standard deviation (error bars) for each group. **, *P* < 0.01. ***, *P* < 0.001

In order to investigate whether PCV2 could activate the RIG-I-like signaling pathway in PIECs, qPCR was employed to measure the expression levels of RIG-I, MDA-5 and MAVS mRNA. Polyriboinsine-polyribocyaidylic acid (Poly(I:C), which could activate RIG-I and MDA-5 to produce IL-8 in mesothelial cells [18] and some epithelial cells [19], was used as the positive control in this study. The expression levels of RIG-I, MDA-5 and MAVS mRNA in PCV2-PIECs group were higher than those in the PIECs group at 12 and 24 h post-infection (hpi) (*P* < 0.05) (Fig. 1a, b, c) while substantially increased at 24 hpi (*P* < 0.01). Western blot showed that the protein expression levels of RIG-I, MDA-5 and MAVS were significantly upregulated in PCV2-PIECs group compared with the PIECs group (*P* < 0.01) (Fig. 1d and Fig. 2). All results above indicated that the RIG-I-like signaling pathway was activated after PCV2 infection.

### PCV2 does not activate NF-κB signaling pathway in PIECs

**Fig. 3:**
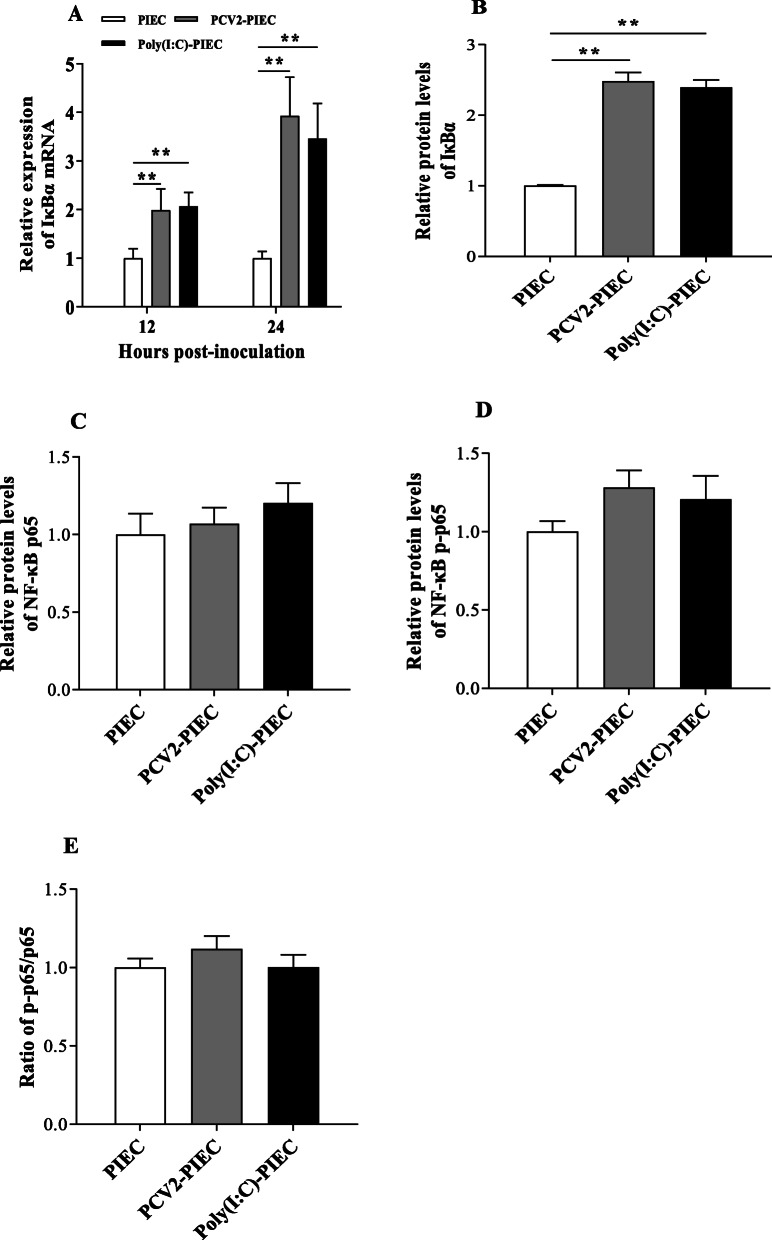
The relative expression levels of key proteins in NF-κB signaling pathway. PIECs were infected with PCV2 (MOI = 0.5) for 1 h and then cultured for 12 and 24 h. The IκBα mRNA expression was assessed by qPCR at two time points (**a**). Quantitative analyses of the immunoblot data of IκBα (**b**), NF-κB p65 (**c**) and NF-κB p-p65 (**d**) was performed at 24 h. The ratio of NF-κB p-p65/ NF-κB p65 (**e**). The uninfected PIEC was used as a negative control and Poly(I:C) treatment was used as a positive control. Data are results of three independent experiments and presented as the mean and standard deviation (error bars) for each group. **, *P* < 0.01

**Fig. 4:**
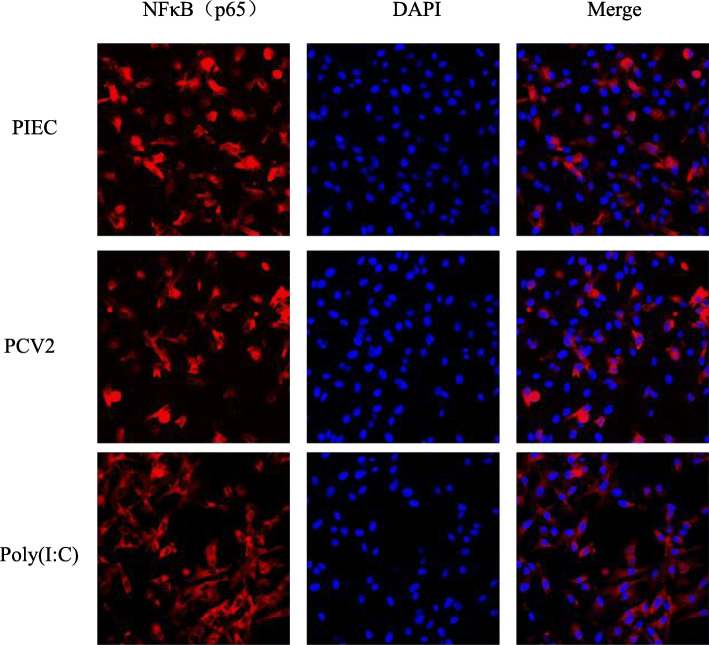
Nuclear translocation of NF-κB after PCV2 infection in PIECs. PIECs were infected with PCV2 (MOI = 0.5) for 1 h and then cultured for 24 h. Fluorescence confocal microscopy was used to measure the cellular localization of NF-κB p65 in PCV2-infected PIECs. The localization of NF-κB p65 (red) was observed with a fluorescence microscope using immunofluorescence staining with anti-NF-κB p65 and Alexa Fluor®647 conjugate anti-mouse IgG. Nuclei were stained with DAPI. The uninfected PIEC was used as a negative control and Poly(I:C) treatment was used as a positive control. Bar = 20 μm

In addition to RIG-I-like signaling pathway, NF-κB signaling pathway also plays an important role in the production of inflammatory-related factors. Based on the previous results [9], we chose to test whether NF-κB signaling pathway was activated in PIECs following PCV2 infection. The qPCR results showed that the mRNA expression level of IκBα was significantly upregulated at 12 and 24 hpi in PCV2-PIECs group (*P* < 0.01) (Fig. 3a). Western blot showed that the protein expression level of IκBα was also upregulated in PCV2-PIECs group (Fig. 1d and Fig. 3b). But the expressions of NF-κB p65 and NF-κB p-p65 had no significant changes (*P* > 0.05) (Fig. 1d and Fig. 3c, d, e). Fluorescence confocal microscopy further confirmed that there was no significant nuclear translocation of NF-κB in PIECs (Fig. 4), suggesting that NF-κB was not activated. All results above indicated that PCV2 did not activate NF-κB signaling pathway in PIECs.

### PCV2 activates JNK signaling pathway in PIECs

**Fig. 5:**
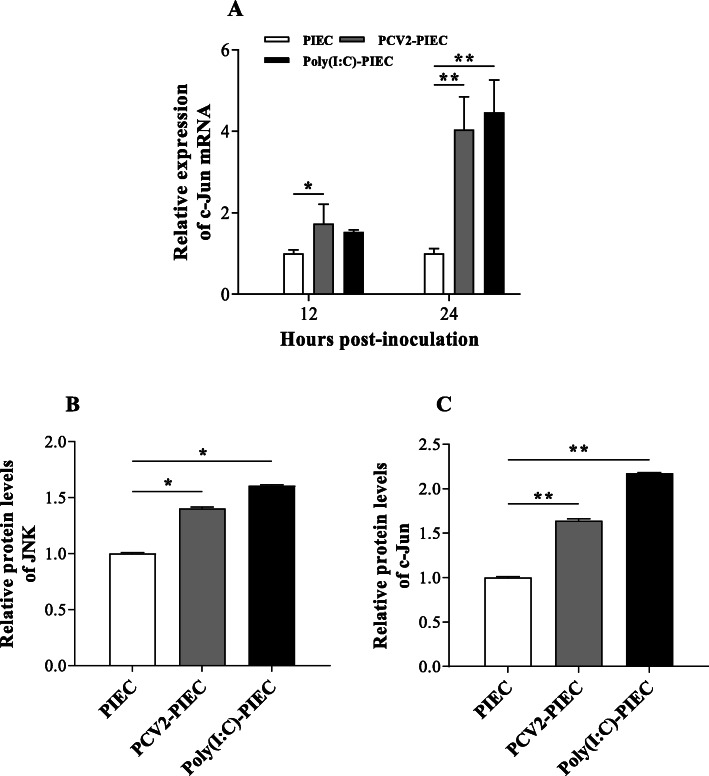
The relative expression levels of key proteins in JNK signaling pathway. PIECs were infected with PCV2 (MOI = 0.5) for 1 h and then cultured for 12 and 24 h. The c-Jun mRNA expression was assessed by qPCR at two time points (**a**). Quantitative analyses of the immunoblot data of JNK (**b**) and c-Jun (**c**) was performed at 24 h. The uninfected PIEC was used as a negative control and Poly(I:C) treatment was used as a positive control. Data are results of three independent experiments and presented as the mean and standard deviation (error bars) for each group. *, *P* < 0.05. **, *P* < 0.01

**Fig. 6:**
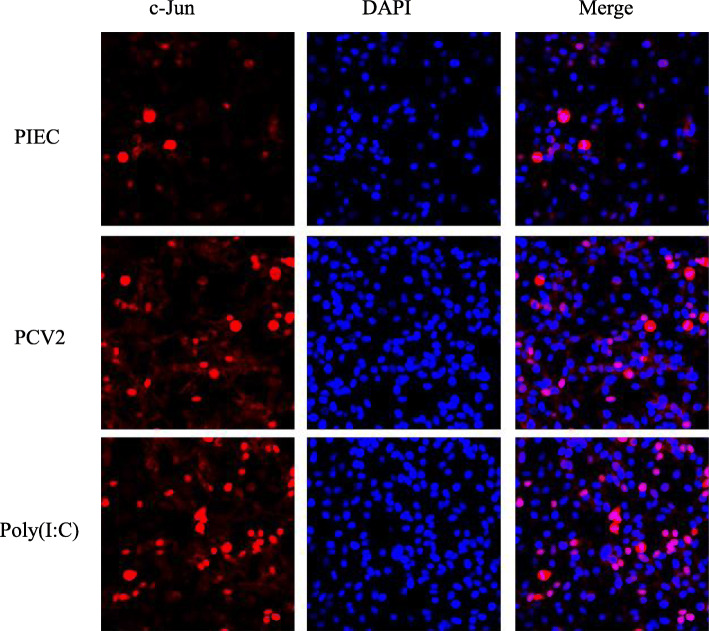
Nuclear translocation of JNK after PCV2 infection in PIECs. PIECs were infected with PCV2 (MOI = 0.5) for 1 h and then cultured for 24 h. Fluorescence confocal microscopy was used to measure the cellular localization of c-Jun in PCV2-infected PIECs. The localization of c-Jun (red) was observed with a fluorescence microscope using immunofluorescence staining with anti-c-Jun and Alexa Fluor®647 conjugate anti-rabbit IgG. Nuclei were stained with DAPI. The uninfected PIEC was used as a negative control and Poly(I:C) treatment was used as a positive control. Bar = 20 μm

JNK signaling pathway is an important part of MAPK pathway, which plays an important role in cell cycle, reproduction, apoptosis and cell stress. Based on our microarray results (unpublished data), whether JNK signaling pathway could be activated in PIECs infected with PCV2 were investigated. The mRNA expression levels of c-Jun in the PCV2-PIECs at two time points were both higher than those in the PIECs group (*P* < 0.05) (Fig. 5a). The protein levels of JNK and c-Jun in the PCV2-PIECs group were both higher than those in the PIECs group at 24 hpi (*P* < 0.05) (Fig. 1d and Fig. 5b, c). In the fluorescence confocal experiment, a significant nuclear translocation of c-Jun in the PCV2-PIECs group was observed as compared to the control group (Fig. 6). All indicated that JNK signaling pathway in PIECs was activated after PCV2 infection.

### RIG-I/MDA-5/MAVS is involved in the upregulation of endothelial-derived IL-8 expression induced by PCV2 in PIECs

**Fig. 7:**
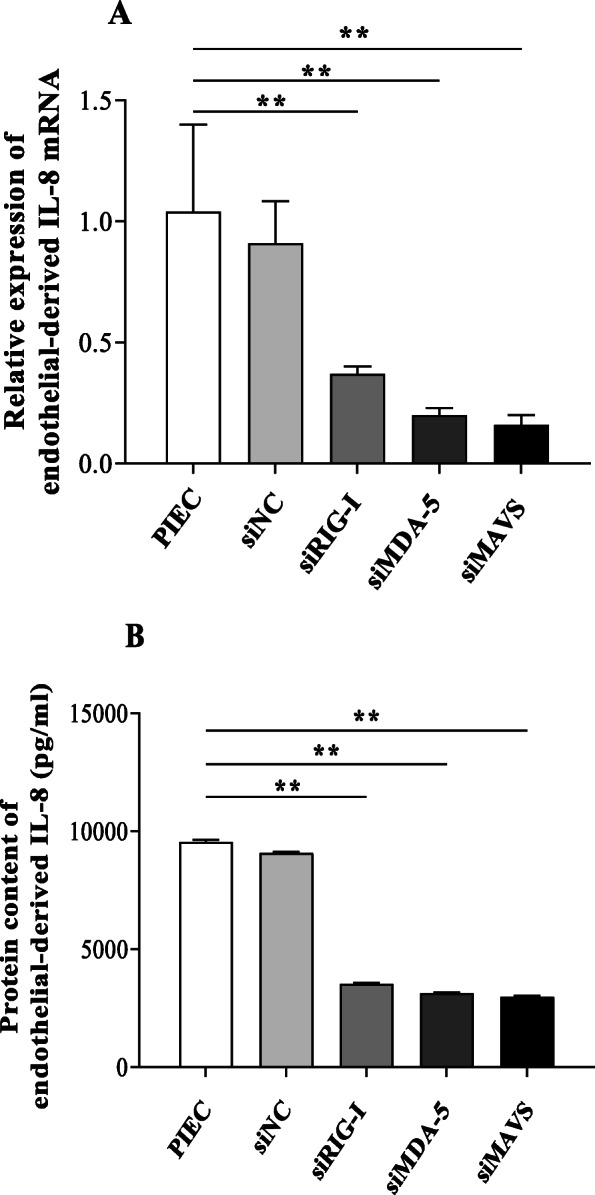
Changes in endothelial-derived IL-8 mRNA and protein expression in PCV2 infected PIECs after the knockdown of RIG-I, MDA-5 or MAVS. PIECs were transfected with siRIG-I, siMDA-5, siMAVS or siNC for 24 h. Afterwards, all groups were infected with PCV2 (MOI = 0.5) for 1 h and then cultured for 24 h. The expression level of endothelial-derived IL-8 mRNA was measured by qPCR (**a**) and the protein was measured by ELISA (**b**). siNC treatment was used as a negative control and untransfected PIEC was used as a blank control. Data are results of three independent experiments and are represented as the mean and standard deviation (error bars) for each group. **, *P* < 0.01

To study the role of RIG-I/MDA-5/MAVS in the production of endothelial-derived IL-8 induced by PCV2, siRNA was designed to knockdown the expression of RIG-I, MDA-5 or MAVS. The expression levels of endothelial-derived IL-8 mRNA and protein in the PCV2-siRIG-I group were significantly downregulated compared to those in the PCV2-PIECs group (*P* < 0.01) (Fig. 7). Similar results were also obtained in the PCV2-siMDA-5 and the PCV2-siMAVS groups (Fig. 7). These results strongly suggested that the RIG-I/MDA-5/MAVS pathway was involved in the upregulation of endothelial-derived IL-8 expression induced by PCV2 in PIECs.

### JNK signaling pathway contributes to the upregulation of endothelial-derived IL-8 expression induced by PCV2 in PIECs

**Fig. 8:**
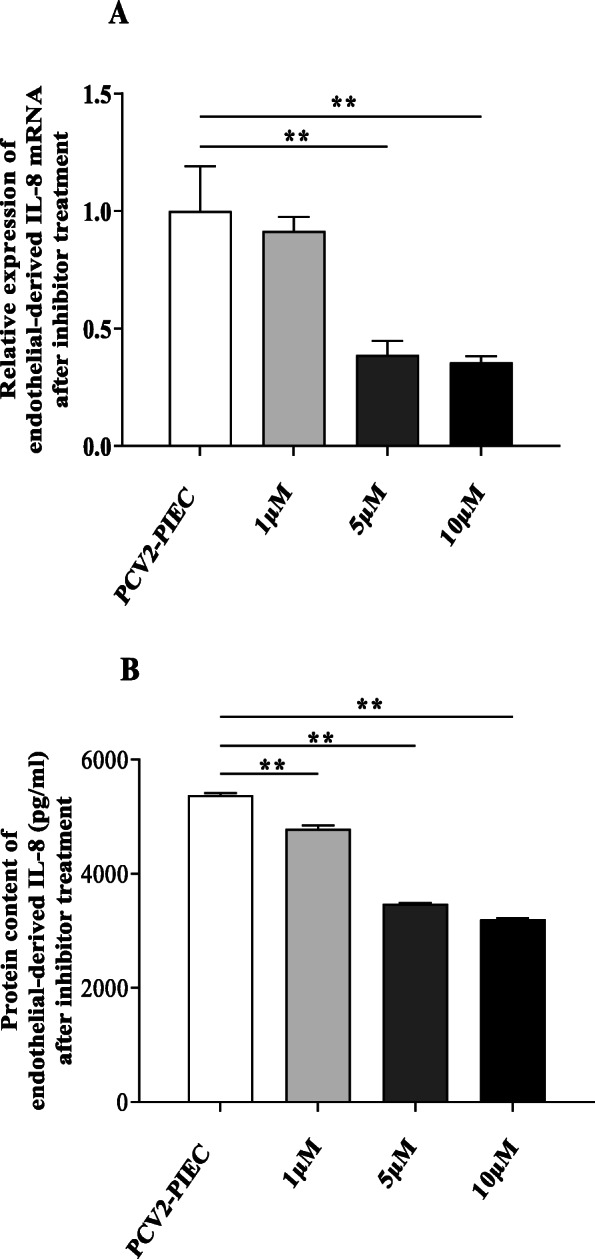
Changes in endothelial-derived IL-8 mRNA and protein expression in PCV2 infected PIECs after JNK inhibitor treatment. PIECs were infected with PCV2 (MOI = 0.5) after 1 h of JNK inhibitor treatment with different concentrations (1 μM, 5 μM and 10 μM) and then cultured for 24 h. The level of endothelial-derived IL-8 mRNA expression was measured by qPCR (**a**) and the protein expression was measured by ELISA (**b**). The PCV2 infected PIECs without the inhibitor treatment was used as a negative control. Data are results of three independent experiments and are represented as the mean and standard deviation (error bars) for each group. **, *P* < 0.01

To determine whether the expression of endothelial-derived IL-8 induced by PCV2 was regulated by JNK signaling pathway, a JNK inhibitor was used to treat PIECs. The expression level of endothelial-derived IL-8 in the inhibitor treatment group was significantly downregulated compared to the control group (*P* < 0.01) (Fig. 8). It suggested that JNK pathway contributed to the upregulation of endothelial-derived IL-8 expression induced by PCV2 in PIECs.

## Discussion

RIG-I and MDA-5, the sensory molecules of viral infection [20, 21], can recognize RNA viruses and some DNA viruses by RNA polymerase III [22] and induce the production of different cytokines by identifying exogenous viral molecules [21, 23]. RIG-I mainly recognizes Paramyxoviridae (Newcastle Disease Virus, Sendai Virus, and Respiratory Syncytial Virus) [20], Flavivirus [24] and Rhabdoviridae (Rabies Virus) [25]. MDA-5 mainly recognizes Coronaviridae (Mouse Hepatitis Virus) [26]. RIG-I and MDA-5 can simultaneously recognize Measles Virus [27] and West Nile Virus [28]. In both VR1BL and PK-15 cells, RIG-I and MDA-5 also recognize PCV2 together [15, 29], which was similar to that of our study in PIECs. All these indicated that PCV2 could infect different kinds of cells in piglets via RIG-I/MDA-5 pathway.

MAVS, which is located on the outer membrane of mitochondria, is a key common adaptor of retinoic acid-induced gene I-like receptors (RLRs) [30]. Caspase recruitment domains (CARDs) are released from the signal suppression state and recruit MAVS by CARD-CARD interaction after RIG-I and MDA-5 identify the viral components in the cytoplasm [31–33]. The activation of MAVS can effectively promote the downstream signaling pathway and induce the productions of interferon and proinflammatory factor [34]. Once MAVS is degraded, its downstream signal will also be blocked [35, 36]. In this study, we confirmed that PCV2 could activate the adapter protein MAVS in PIECs. siRNA (siRIG-I, siMDA-5, siMAVS) transfection experiments further confirmed the roles of these signaling pathway above in the production of endothelial-derived IL-8 in PIEC after PCV2 infection. Taken together, PCV2 could activate the signaling pathway of RIG-I/MDA-5/MAVS in PIECs and therefore induced the production of endothelial-derived IL-8.

Based on the previous results [9] and microarray results (unpublished data), we chose to investigate whether NF-κB and JNK signaling pathway were the key downstream pathway in this process. The results demonstrated that PCV2 infection could activate JNK but not NF-κB signaling pathway in PIECs. It indicated that PCV2 could activate JNK signaling pathway to upregulate the production of endothelial-derived IL-8 in PIECs, which was different from the mechanism of monocyte-derived IL-8 production in downstream pathway.

## Conclusions

**Fig. 9:**
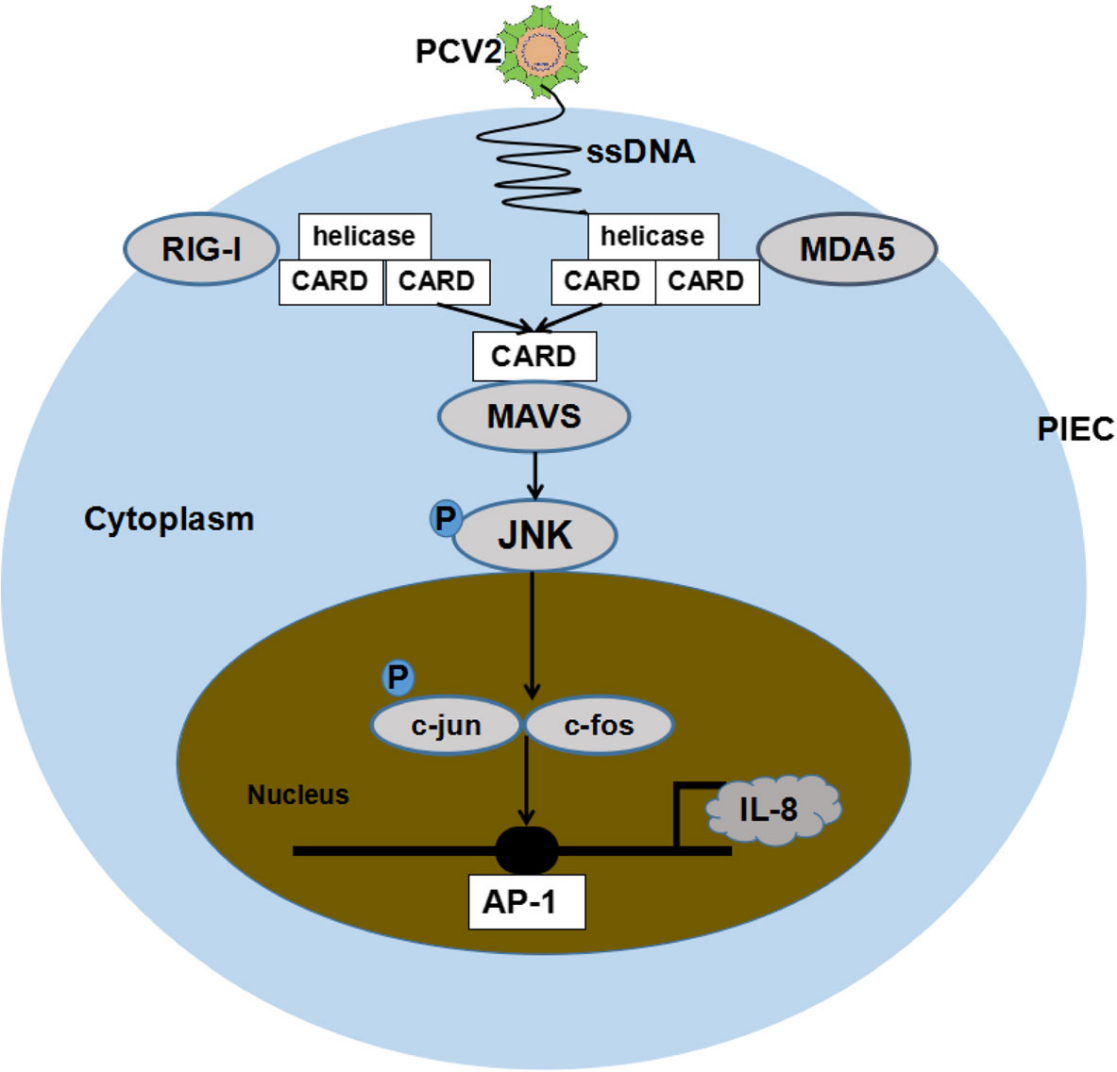
Model of endothelial-derived IL-8 production induced by PCV2 in PIECs. Identification of PCV2 by RIG-I and MDA-5 activated a cascade of changes that led to the upregulation of MAVS and then stimulated the nuclei accumulation of JNK. As a result, endothelial-derived IL-8 was apparently upregulated. Knockdown of RIG-I, MDA-5 or MAVS using siRNA resulted in the negative modulation of endothelial-derived IL-8 secretion. Similar results were also obtained by the JNK inhibitor treatment. It shows that PCV2 can upregulate the production of endothelial-derived IL-8 in PIECs via the RIG-I/MDA-5/MAVS/JNK signaling pathway

Our study demonstrated that PCV2 could upregulate the production of endothelial-derived IL-8 via RIG-I/MDA-5/MAVS/JNK signaling pathway in PIECs (Fig. 9). It elucidated the mechanism of endothelial-derived IL-8 production in PCV2 infected endothelial cells, which was different from the mechanism of monocyte-derived IL-8 production. Our study provides an insight into the further study of endothelial dysfunction and vascular system disorder caused by PCV2.

## Methods

### Cells and virus

Porcine iliac arterial endothelial cell (PIEC) (Cat. No. GN015) was purchased from the Cell Resource Center of the Shanghai Institute of Biological Sciences (Shanghai, China). The cells were cultured in RPMI 1640 medium (GIBCO, Grand Island, NY, USA) supplemented with 10% fetal bovine serum (FBS) (BI, Kibbutz Beit, Israel), and incubated in an atmosphere of 5% CO_2_ at 37 °C (Thermo Fisher Scientific, Waltham, MA, USA). The PCV2 (PCV2-SD/2008, GenBank accession number: GQ174519; it was isolated and identified by Animal Infectious Disease Laboratory of Hebei Agricultural University) stock was prepared in PK-15 cells with a titer of 10^5.5^ TCID_50_/ml.

### Extraction of total RNA and quantitative PCR

PCV2 (MOI = 0.5) was used to infect 70% confluent PIECs in 6-well plate (5 × 10^5^ cells/well). Cells were incubated for 1 h in an atmosphere of 5% CO_2_ at 37 °C, and RPMI1640 supplemented with 10% FBS was then added to infected cells. The experiment was performed with the control group (PIECs), the positive control group (Poly(I:C)-PIECs) and the infection group (PCV2-PIECs). Cell samples were harvested with Trizol (Invitrogen, Carlsbad, CA, USA) at 12 and 24 hpi. Total RNA was extracted according to the instructions of the RNA extraction kit (Aidlab, Beijing, China), and the extracted RNA was reverse transcribed to synthesize cDNA according to the reverse transcription kit (Cwbio, Beijing, China). The mRNA expression of each molecule (RIG-I, MDA-5, MAVS, IκBα, c-Jun, endothelial-derived IL-8) was detected by qPCR. The reactions were performed in triplicate using SYBR Green Real-time PCR Master Mix (TOYOBO, Osaka, Japan) in the Mx3005P Detection System (Genetimes, Shanghai, China). The total reaction system was 20 μl and contained the following: SYBR® Green Real-time PCR Master Mix (10 μl), 25 pmol/μl upstream and downstream primers (each for 0.2 μl), template DNA (1.0 μl), and water (8.6 μl). The conditions were as follows: 95 °C for 5 min, followed by 40 cycles of 95 °C for 40 s, 56 °C for 30 s, and 72 °C for 30 s. Water was used to replace template DNA in the negative control. All results were analyzed using MxPro Software. Primer sequences for qPCR are listed in Table 1.

**Table 1:** Primers used for qPCR

Genes Source/ accession No.	Accession number	Primer sequence (5′-3′)	Annealing temperature (°C)	Products (bp)
RIG-I	NM_213804.2	For: TCTGGAGCAACGTCTGACAC	56	166
		Rev: GTTTGCTGGTGTTGTGGCAT		
MDA-5	NM_001100194.1	For: TGGACACAGCAGTGAGTTCAAGC	51	105
		Rev: GCCACCGTGGTAGCGATAAGC		
MAVS	XM_005672763.1	For: GGCATCAGAAGCAGGACACAGAAC	59	177
		Rev: CAGTGGAGGAGGAGGCAGTAGAC		
IκBα	NM_001005150.1	For: CCAACCAGCCAGAAATCGCT	58	168
		Rev: GCAGAATGGAGTGGAGGTGC		
c-Jun	NM_213880.1	For: GCATCGCTGCCTCCAAGT	56	224
		Rev: CCCAACAGTCTCGCCTCAAA		
IL-8	NM_213867	For: TCCTGCTTTCTGCAGCTCTC	59	100
		Rev: GGGTGGAAAGGTGTGGAATG		
β-actin	XM_003357928	For: TCATCACCATCGGCAACT	58	133
		Rev: TTCCTGATGTCCACGTCGC		

Note: β-actin was used as the internal control

*bp* Base pairs, *For* Forward primer, *Rev*. Reverse primer

### Reagents and antibodies

Poly(I:C) (InvivoGen, San Diego, CA, USA) was used for the positive control. All antibodies used for Western blot, such as primary antibodies (RIG-I, MDA-5, MAVS, IκBα, p65, p-p65, JNK, c-Jun, β-actin), HRP-conjugated anti-mouse secondary antibody and HRP-conjugated anti-rabbit secondary antibody were purchased from Cell Signaling Technology (CST, Danvers, MA, USA). Alexa Fluor®647 conjugated anti-mouse IgG (CST, Danvers, MA, USA) and Alexa Fluor®647 conjugated anti-rabbit IgG (Beyotime, Shanghai, China) were used for the fluorescence confocal microscopy. JNK inhibitor SP600125 was purchased from Beyotime, Shanghai, China and Protein Marker 26616 was purchased from Thermo Fisher Scientific, Waltham, MA, USA.

### Western blot analysis

70% confluent PIECs in a 6-well plate was infected with PCV2 (MOI = 0.5) with Poly(I:C) (5 μg/ml) for 1 h in an atmosphere of 5% CO_2_ at 37 °C. The cells were further incubated for 24 h in RPMI1640 supplemented with 10% FBS. Total proteins were extracted from the collected cells according to the manufacturer instructions of total protein extraction kit (Keygen, Nanjing, China) and quantified by BCA protein assay kit (Cwbio, Beijing, China). Quantitative protein was subjected to SDS-PAGE and then transferred onto a polyvinylidene difluoride (PVDF) membrane. The membrane was further blocked with Tris Buffer Solution Tween-20 (TBST) containing 5% BSA for 2 h and then incubated overnight in a 1:2000 dilution of primary antibody at 4 °C with gentle shaking. After washed four times in TBST, the membrane was blotted with the second antibody (1:5000) for 1 h. Actin was used as the internal reference. The blot was visualized with Omega Aplegen scanner (Aplegen, San Francisco, CA, USA). The gray matter volume of protein was quantified by importing the images into an ImageJ analysis software. The original blots can be referred in the Additional file 1.

### Fluorescence confocal microscopy

The cells were treated in the same manner as Western blot analysis. At 24 hpi, the cells were digested with 0.25% trypsin. All cells were centrifuged and the supernatant was discarded. The cells were suspended in 200 μl of RPMI1640 supplemented with 10% FBS, and then dripped into a confocal chamber treated with polylysine. After adhering for 4 h, the supernatant was discarded, PIECs were fixed with 2% paraformaldehyde for 15 min at room temperature. After washed three times with phosphate-buffered saline (PBS), they were blocked with blocking solution for 1 h. Then, the diluted primary antibody (p65 1:800, c-Jun 1:400) was added and incubated overnight at 4 °C. The diluted secondary antibody (1:500) was added in the dark and incubated at room temperature for 1 h. The nucleus was stained with DAPI solution at 37 °C for 20 min. Anti-fluorescence attenuating seals were added dropwise protected from light at 4 °C. The cells were observed with a laser scanning confocal microscopy (Olympus FV100, Hamburg, Germany).

### siRNA interference

5 μl of Lipofectamine 2000 (Invitrogen, Carlsbad, CA, USA) and 150 pmol of siRNA (siRIG-I, siMDA-5, siMAVS) (Gene Pharma, Shanghai, China) were added into 6-well plates with pre-plated PIECs in serum-free RPMI1640. After 6 h, the serum-free medium was replaced by RPMI1640 supplemented with 10% FBS. For optimal knockdown, the transfection efficiency of siRIG-I, siMDA-5, and siMAVS were detected by qPCR after 24 h and Western blot after 48 h (Additional file 2). The siRNA primers were listed in Table 2.

**Table 2:** siRNA primer sequences used for cell transfection

Genes Source/ accession No.	Accession number	Primer sequence (5′-3′)
siRIG-I	EU126659.1	For: CACCAGCAAACAGCAUCCUUAUAAU
		Rev: AUUAUAAGGAUGCUGUUUGCUGGUG
siMDA-5	EU006039.1	For: GCAGACGAAGUUUGCUGACUAUCAA
		Rev: UUGAUAGUCAGCAAACUUCGUCUGC
siMAVS	AB287431.1	For: AUGGAUAGCCAGCCUUUCUTT
		Rev: AGAAAGGCUGGCUAUCCAUCC
siNC		For: GCGCGCUUUGUAGGAUUCGTT
		Rev: CGAAUCCUACAAAGCGCGCTT

*For* Forward primer, *Rev*. Reverse primer

### Inhibitor treatment

PIECs were inoculated into 6-well plates. When the cells covered 50–70% of the surface, they were treated with JNK inhibitor SP600125. The same dose of dimethyl sulfoxide (DMSO) was used as the control (The final concentration of DMSO was less than 0.1%). After 1 h of treatment, the cells were washed with PBS and then infected with PCV2 (MOI = 0.5) for 1 h. The cells and supernatants were sampled after 24 h. The expression of endothelial-derived IL-8 was detected by qPCR and ELISA.

### ELISA

The expression of endothelial-derived IL-8 in the supernatant of each group was determined according to the manufacturer instructions of porcine IL-8 ELISA kit (CUSABIO, Wuhan, China). The OD value was read at 490 nm using a microplate reader (BioTek, Winooski, VT, USA). Standard curves were obtained according to Curve Expert software using the dilutions of a sample from the kit as the template. Concentration of the samples in the different groups was calculated according to the standard curve.

### Statistical analysis

We performed all statistical analyses using the SPSS Statistics 17 (IBM Corporation). The results are expressed as the mean ± standard deviation (SD). Differences between the experimental groups and the control groups were analyzed using *ANOVA* followed by the Duncan’s multiple range test for multiple comparisons. *P* < 0.05 was considered to be statistically different and was denoted by *. *P* < 0.01 was considered to be significantly different and was denoted by **. *P* < 0.001 was considered to be extremely significantly different and was denoted by ***. Unless indicated otherwise, the experiments were performed in triplicate (*n* = 3).

## Supplementary information

**Additional file 1.** The original blots for the figures. For our gel data, the target proteins analyzed by western blot were transferred from SDS-PAGE and the PVDF membrane would be sliced into different strips according to their protein size identified with the loading marker. Then the panel of antibodies targeting these proteins were used for western blot analysis. Our SOP for processing the gel data only collected the pictures with target protein regions instead of the whole gel, so here are the original gels for the data we presented in the manuscript.

**Additional file 2 **The transfection efficiency of siRIG-I, siMDA-5 and siMAVS. PIECs were transfected with siRIG-I, siMDA-5, siMAVS or siNC for 24 h. Afterwards, all groups were infected with PCV2 (MOI = 0.5) for 1 h and then cultured for 24 h. The result of qPCR showed the mRNA level of RIG-I, MDA-5 or MAVS at 24 h (A). The result of Western blot showed the protein level of RIG-I (B), MDA-5 (C) or MAVS (D) at 48 h. siNC treatment was used as a negative control and untransfected PIEC was used as a blank control. Data are results of three independent experiments and are represented as the mean and standard deviation (error bars) for each group. **, *P* < 0.01.

## Data Availability

The datasets used and/or analysed during the current study are available from the corresponding author on reasonable request.

## Abbreviations

*PCVD*: Porcine circovirus disease
*PCV2*: Porcine circovirus type 2
*PIECs*: Porcine iliac artery endothelial cells
*PMWS*: Postweaning multisystemic wasting syndrome
*RLRs*: Retinoic acid-induced gene I-like receptors
*RIG-I*: Retinoic acid-inducible gene I
*MDA-5*: Melanoma differentiation-associated gene-5
*PRRs*: Pattern recognition receptors
*Poly(I:C)*: Polyriboinsine-polyribocyaidylic acid
*MAVS*: Mitochondrial antiviral signaling protein
*Hpi*: Hours post-infection
*FBS*: Fetal bovine serum
*PVDF*: Polyvinylidene difluoride
*TBST*: Tris buffer solution tween-20
*PBS*: Phosphate-buffered saline
*DMSO*: Dimethyl sulfoxide
*SD*: Standard deviation
