# Supplementary material for: Porcine circovirus type 2 upregulates endothelial-derived IL-8 production in porcine iliac artery endothelial cells via the RIG-I/MDA-5/MAVS/JNK signaling pathway
Source: BMC Vet Res. 2020 Jul 29;16:265. doi: 10.1186/s12917-020-02486-1 (PMC7392700; doi:10.1186/s12917-020-02486-1)

**Additional file 1. The original blots for the figures**

**Note:** For our gel data, the target proteins analyzed by western blot were transferred from SDS-PAGE and the PVDF membrane would be sliced into different strips according to their protein size identified with the loading marker. Then the panel of antibodies targeting these proteins were used for western blot analysis. Our SOP for processing the gel data only collected the pictures with target protein regions instead of the whole gel, so here are the original images of all repeat experiments that were performed for the different samples in the manuscript.

**The first result**

**IκBα**

**39kd**


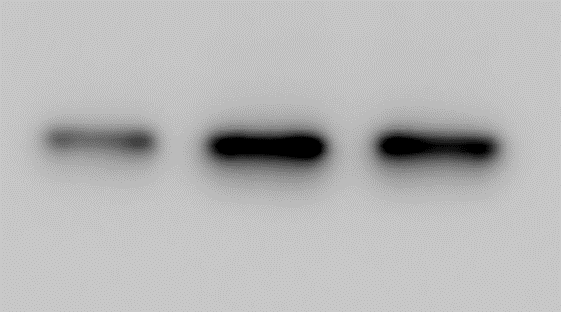


**MAVS**

**52kd**


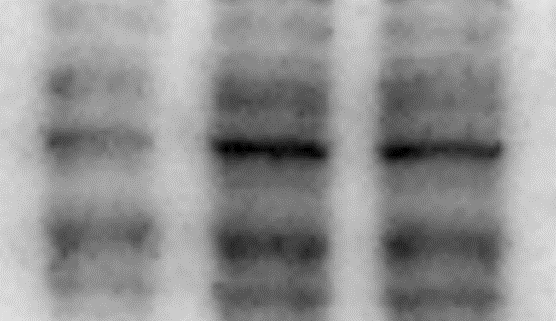


**140kd**

**MDA-5**


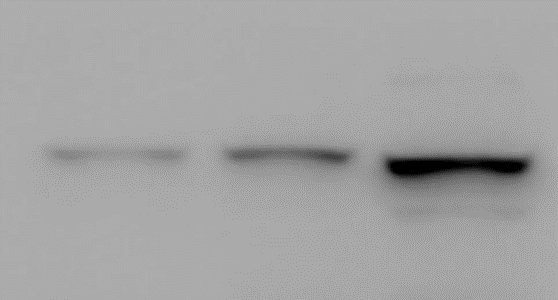


**110kd**

**RIG-I**

**PIEC PCV2 Poly(I:C)**


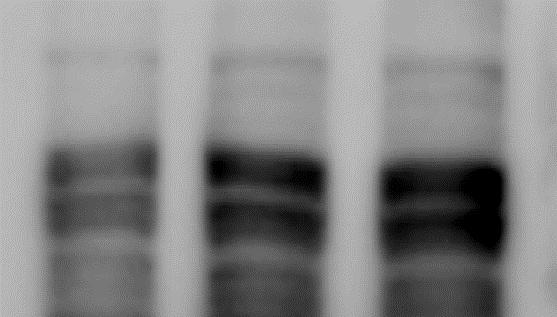


**β-actin**

**42kd**


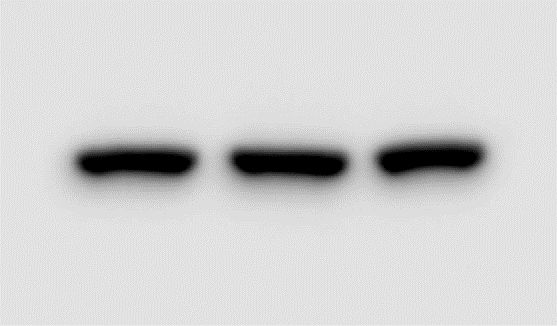


**c-Jun**

**43kd**


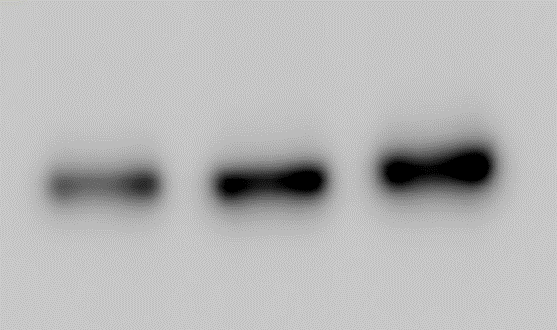


**JNK**

**54kd**


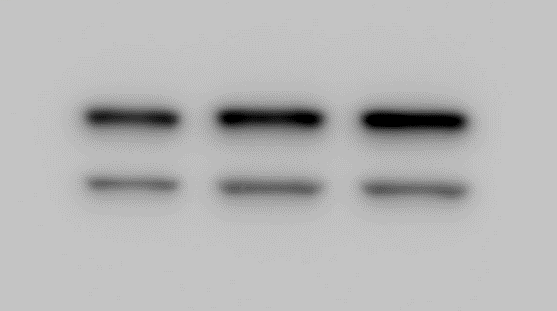


**p-p65**

**65kd**


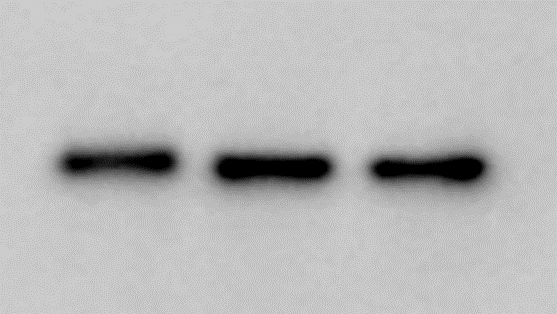


**p65**

**65kd**


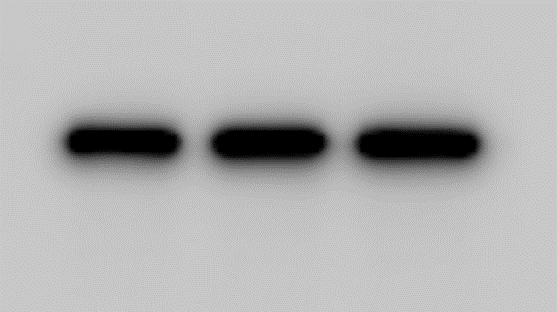


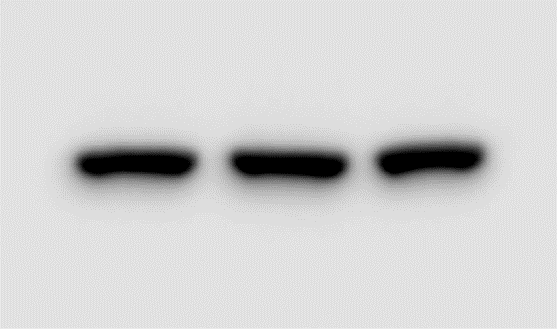


**The second result**

**p65**

**IκBα**

**MAVS**

**52kd**

**39kd**

**65kd**


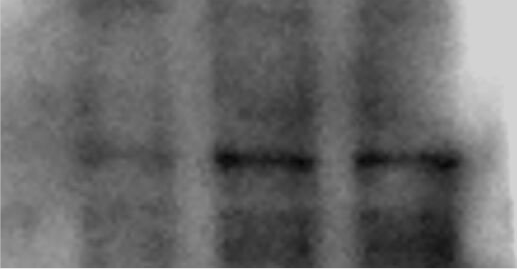


**MDA-5**

**140kd**

**RIG-I**

**110kd**

**PIEC PCV2 Poly(I:C)**


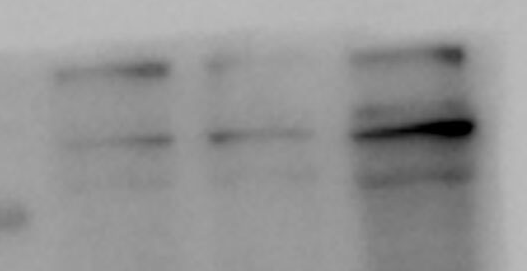

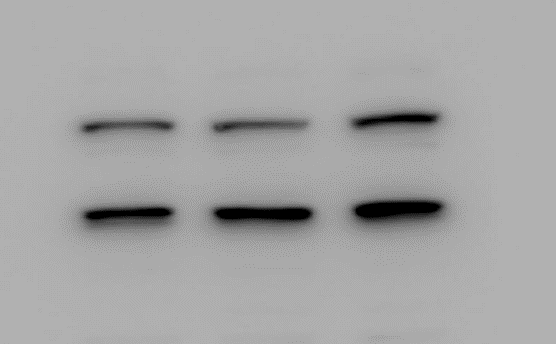

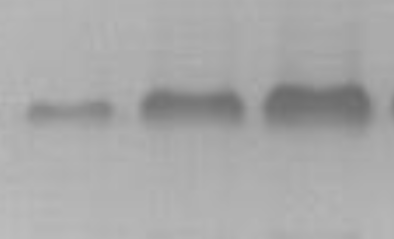

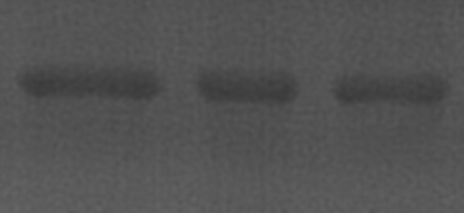


**c-Jun**

**JNK**

**p-p65**

**42kd**

**43kd**

**54kd**


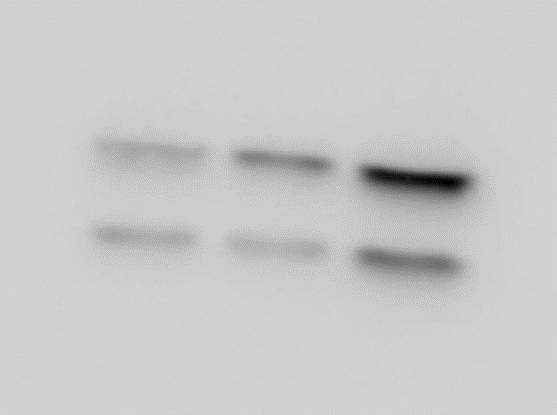

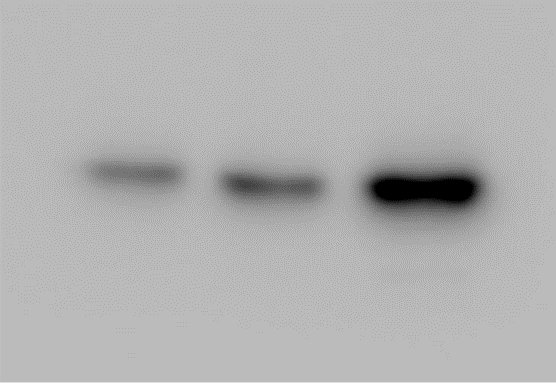


**β-actin**


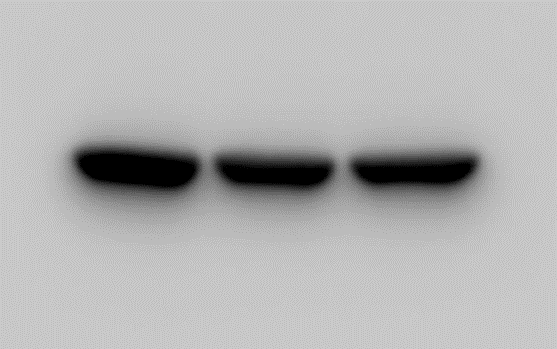

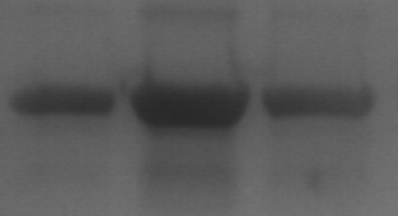


**65kd**

**The third result**

**110kd**

**PIEC PCV2 Poly(I:C)**

**RIG-I**


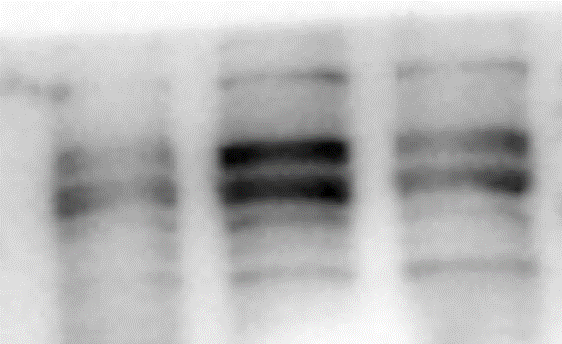


**65kd**

**p65**

**39kd**

**IκBα**

**52kd**

**MAVS**

**140kd**

**MDA-5**


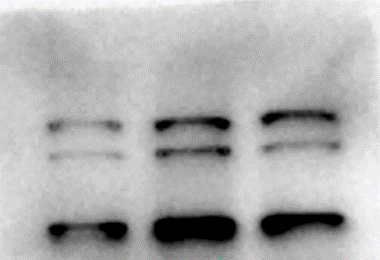

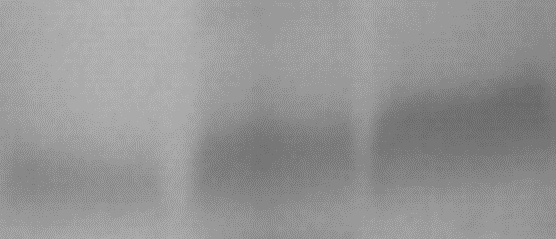


**65kd**

**p-p65**


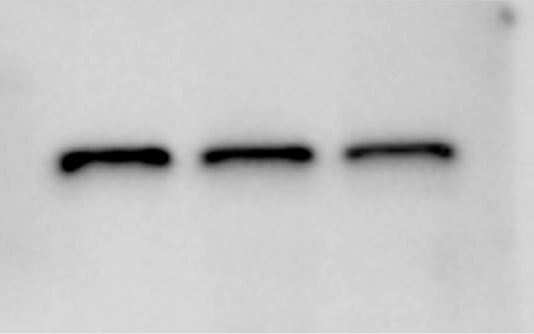


**JNK**

**54kd**


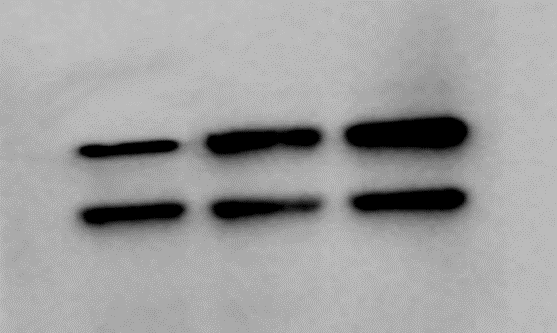

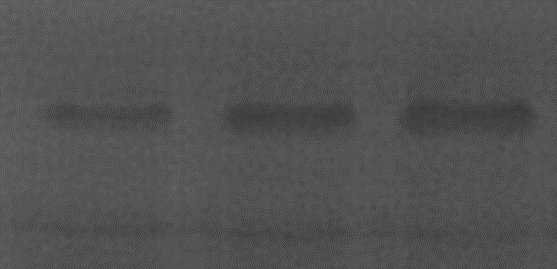

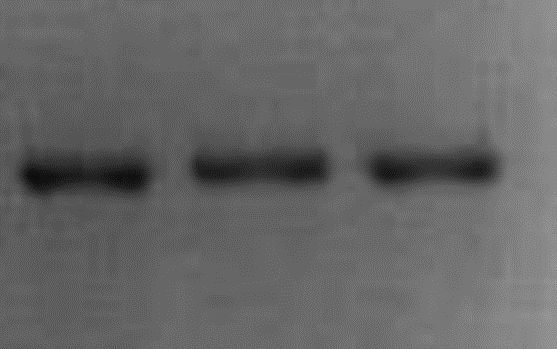


**c-Jun**

**43kd**

**β-actin**

**42kd**

**43kd**

**c-Jun**

**42kd**

**β-actin**


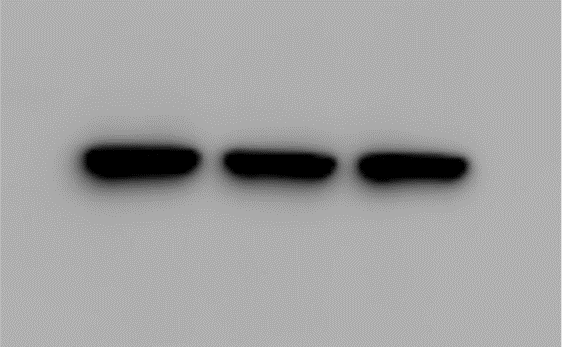

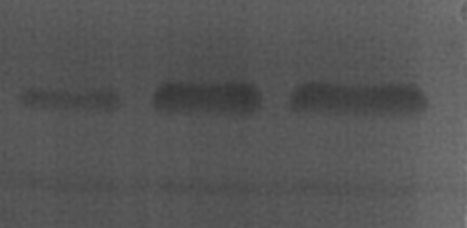

Supplement: Supplementary file 1 — Additional file 1. The original blots for the figures. For our gel data, the target proteins analyzed by western blot were transferred from SDS-PAGE and the PVDF membrane would be sliced into different strips according to their protein size identified with the loading marker. Then the panel of antibodies targeting these proteins were used for western blot analysis. Our SOP for processing the gel data only collected the pictures with target protein regions instead of the whole gel, so here are the original gels for the data we presented in the manuscript. [file 12917_2020_2486_MOESM1_ESM.docx]
