# Supplementary material for: Porcine circovirus type 2 upregulates endothelial-derived IL-8 production in porcine iliac artery endothelial cells via the RIG-I/MDA-5/MAVS/JNK signaling pathway
Source: BMC Vet Res. 2020 Jul 29;16:265. doi: 10.1186/s12917-020-02486-1 (PMC7392700; doi:10.1186/s12917-020-02486-1)
Supplement: Supplementary file 2 — Additional file 2 The transfection efficiency of siRIG-I, siMDA-5 and siMAVS. PIECs were transfected with siRIG-I, siMDA-5, siMAVS or siNC for 24 h. Afterwards, all groups were infected with PCV2 (MOI = 0.5) for 1 h and then cultured for 24 h. The result of qPCR showed the mRNA level of RIG-I, MDA-5 or MAVS at 24 h (A). The result of Western blot showed the protein level of RIG-I (B), MDA-5 (C) or MAVS (D) at 48 h. siNC treatment was used as a negative control and untransfected PIEC was used as a blank control. Data are results of three independent experiments and are represented as the mean and standard deviation (error bars) for each group. **, P < 0.01. [file 12917_2020_2486_MOESM2_ESM.doc]

**B**

β-actin

RIG-Ⅰ

42kd

110kd

PIEC siNC siRIG-Ⅰ


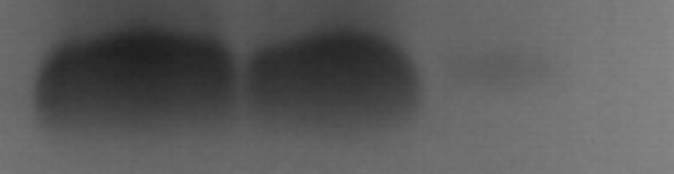

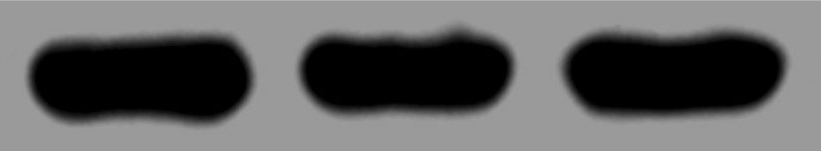


**C**

β-actin

MDA-5

42kd

140kd

PIEC siNC siMDA-5


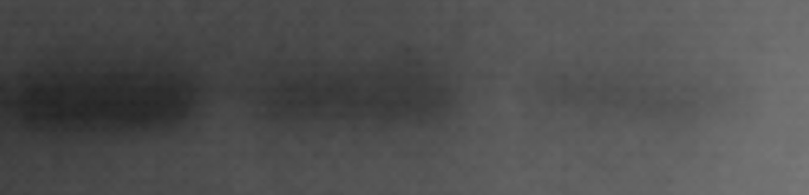

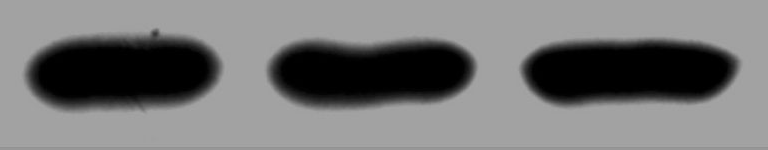


**D**

PIEC siNC siMAVS

β-actin

MAVS

42kd

52kd


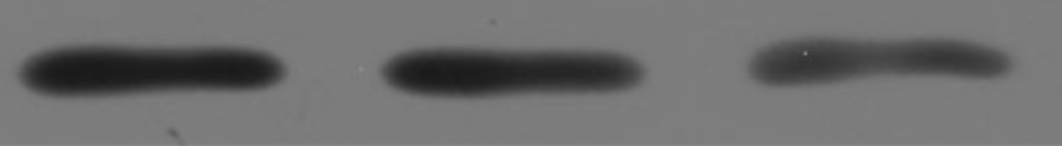

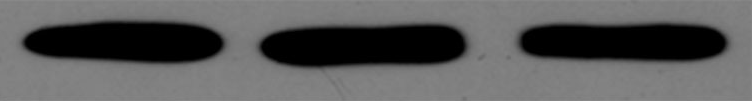


**Additional file 2. The transfection efficiency of siRIG-I, siMDA-5 and siMAVS.** PIECs were transfected with siRIG-I, siMDA-5, siMAVS or siNC for 24 h and then infected with PCV2 (MOI=0.5) for 24 h. The result of qPCR showed the mRNA level of RIG-I, MDA-5 or MAVS at 24 h (A). The result of Western blot showed the protein level of RIG-I (B), MDA-5 (C) or MAVS (D) at 48 h. siNC treatment was used as a negative control and untransfected PIEC was used as a blank control. Data are results of three independent experiments and are represented as the mean and standard deviation (error bars) for each group. **, P < 0.01.
